# Iridoids from *Himatanthus sucuuba* Modulate Feeding Behavior of *Lutzomyia longipalpis*: Integrated Experimental and Computational Approaches

**DOI:** 10.3390/molecules30193937

**Published:** 2025-10-01

**Authors:** Maíra M. H. Almeida, Jefferson D. da Cruz, Maria Athana M. Silva, Samara G. Costa-Latgé, Bruno Gomes, Fernando A. Genta, Jefferson R. A. Silva, Ana Claudia F. Amaral

**Affiliations:** 1 Laboratório de Produtos Naturais e Derivados, Departamento de Produtos Naturais, Farmanguinhos/Fiocruz, Manguinhos, Rio de Janeiro 21041-250, RJ, Brazil; maira-haddad@hotmail.com (M.M.H.A.); jefferson_dacruz@hotmail.com (J.D.d.C.); maria.mpalantinos@fiocruz.br (M.A.M.S.); 2 Programa de Pós-Graduação Acadêmica em Pesquisa Translacional em Fármacos e Medicamentos, Farmanguinhos/Fiocruz, Manguinhos, Rio de Janeiro 21041-250, RJ, Brazil; 3 Laboratório de Bioquímica e Fisiologia de Insetos and INERU, Instituto Oswaldo Cruz, IOC/Fiocruz, Rio de Janeiro 21040-360, RJ, Brazil; samara.latge@gmail.com (S.G.C.-L.); gomesb.phd@gmail.com (B.G.); genta@ioc.fiocruz.br (F.A.G.); 4 Laboratório de Cromatografia, Departamento de Química, Instituto de Ciências Exatas, Universidade Federal do Amazonas, Manaus 69077-000, AM, Brazil; jrocha_01@yahoo.com.br

**Keywords:** iridoids, Apocynaceae, sand flies, leishmaniasis, sugar diet

## Abstract

Control strategies for leishmaniasis increasingly target sand fly vectors through sugar feeding approaches containing bioactive compounds. This study investigated the behavioral and toxicological effects of the iridoids plumericin and isoplumericin, isolated from *Himatanthus sucuuba*, on *Lutzomyia longipalpis* by integrating computational and experimental approaches focused on gustatory system interactions. The iridoids were purified by column chromatography and characterized by GC-MS. The gustatory receptor A0A1B0CHD5 was structurally characterized through homology modeling, followed by molecular docking and 100 ns molecular dynamics simulations. Behavioral assays evaluated survival, repellency, and feeding preferences using sugar solutions supplemented with an iridoid mixture. Toxicity was assessed in *Drosophila melanogaster* as a non-target organism model. Molecular docking results revealed comparable binding affinities between sucrose (ChemPLP score 57.96) and the iridoids plumericin (49.08) and isoplumericin (47.75). Molecular dynamics simulations confirmed the stability of the ligand–receptor complexes and revealed distinct conformational changes. The iridoids did not affect *L. longipalpis* survival, showed no repellency, and did not reduce sugar feeding acceptance. Preference for the control diet was observed only after continuous exposure (48 h), suggesting involvement of post-ingestive sensory processing. No acute toxicity was observed in *D. melanogaster* (96% survival). These findings demonstrate that iridoids preserve vector feeding behavior and survival while exhibiting low toxicity to non-target organisms, supporting their potential use in gustatory modulation strategies in leishmaniasis vector control without compromising ecological safety.

## 1. Introduction

Leishmaniasis comprises a group of infectious parasitic diseases caused by protozoa of the genus *Leishmania* [1,2]. These diseases are classified according to clinical manifestations as visceral (VL), cutaneous (CL), and mucocutaneous leishmaniasis (MCL) [2,3]. Leishmaniasis occurs in 99 countries across tropical and subtropical regions, with an estimated 600,000 to 1,000,000 new CL cases and 50,000 to 90,000 new VL cases annually [1].

Transmission occurs exclusively via sand flies (Diptera: Nematocera: Psychodidae: Phlebotominae) [4]. These are the only hematophagous insects known to transmit leishmaniasis, with females acquiring infection during blood meals from infected mammals [5]. Approximately 1000 sand fly species or subspecies have been described worldwide [6], and more than 160 are confirmed or potential vectors of various *Leishmania* species. In the Old World, species of the subgenus *Leishmania* are transmitted by sand flies of the genus *Phlebotomus*, whereas in the New World, species of the subgenera *Leishmania* and *Viannia* are transmitted by sand flies of the genus *Lutzomyia* [4].

In the Neotropical region, *Lutzomyia longipalpis* Lutz & Neiva, 1912, is the primary vector of *Leishmania infantum*, the causative agent of visceral leishmaniasis (VL) in the Americas [7,8]. In Brazil, the disease poses a significant public health challenge because of its high fatality rate [7,8]. This species is abundant and highly adaptable to urban environments, facilitating disease spread in human-modified landscapes. It is found both indoors and outdoors, in urban and rural areas, and commonly seeks food sources near breeding sites, often associated with domestic animal shelters [9].

The epidemiological relevance of *L. longipalpis* extends beyond *L. infantum chagasi*. Experimental studies [8,10,11,12,13,14] demonstrate that *L. longipalpis* is a permissive vector capable of sustaining infection and transmitting multiple other *Leishmania* species, including those causing cutaneous leishmaniasis such as *L. amazonensis*, *L. major*, *L. mexicana*, and *L. braziliensis*. This permissiveness broadens its epidemiological role by potentially maintaining and disseminating various clinical forms of the disease [8,14]. From an experimental perspective, a key advantage of this sand fly is its amenability to laboratory colonization. Its successful reproduction under controlled conditions makes *L. longipalpis* a widely used model for studies evaluating insecticidal activity, parasite–vector interactions, physiology, genetics, molecular biology, and in vivo anti-*Leishmania* effects [7].

Vector control strategies must consider sand fly nutritional ecology. Blood feeding is essential for females to support oviposition, but both sexes rely on environmental carbohydrates as an energy source for mating, development, and reproduction [15,16].

Accordingly, several studies have investigated carbohydrate-based sugar feeding strategies to control sand flies, aiming to prevent transmission of leishmaniasis. Most formulations have been designed for insecticidal effects, inducing mortality and reducing vector populations [17,18,19,20,21]. More recently, innovative approaches have examined ingestion of antileishmanial compounds and their effects on uninfected sand fly behavior, seeking selective control of infected vectors. In this context, the Apocynaceae family emerges as a promising source of bioactive metabolites with potential activity against *Leishmania* parasites. Apocynaceae, one of the largest and most representative angiosperm families, comprises approximately 250–550 genera and 3700–5100 species, characterized by abundant latex production [22,23]. This family is rich in secondary metabolites, and many genera, including *Rauvolfia*, *Catharanthus*, *Allamanda*, *Strophanthus*, and *Himatanthus,* have long been used in traditional medicine and remain relevant to the pharmaceutical industry [24].

Within the genus *Himatanthus*, *H. sucuuba* (Spruce ex Müll. Arg.) Woodson is particularly noteworthy for its traditional medicinal uses in Amazonian communities. Also known as sucuuba, true sucuuba, or janaguba, it is distributed across Bolivia, Brazil, Colombia, Ecuador, Guyana, Peru, and Venezuela [25,26]. Phytochemical studies show that *H. sucuuba* is rich in terpenoids including cinnamates, α- and β-amyrin acetates, lupeol, lupeol acetate, plumericin, isoplumericin, plumeride, and isoplumeride, present in its latex, bark, roots, and leaves. Its latex and bark also contain phenolic compounds such as gallic acid, catechol, myricetrin, and quercitrin [27]. This chemical diversity underpins its broad medicinal applications. Traditionally, latex or bark preparations are used orally or as topically to treat inflammation, gastric ulcers, skin conditions, and parasitic diseases in Amazonian folk medicine [27,28]. Supporting its traditional use, studies have confirmed the leishmanicidal and antimicrobial activities of *H. sucuuba* latex [29,30,31,32].

Among the bioactive constituents of *H. sucuuba*, the iridoids isoplumericin and plumericin stand out for their antileishmanial properties [33,34]. Given their proven activity, these iridoids represent promising candidates for incorporation into sand fly diets to evaluate survival, feeding behavior, and biological effects in *Lutzomyia longipalpis*. Such studies may elucidate interactions with the sensory systems of these vectors.

This study investigated these effects through an integrated experimental and computational approach. Behavioral parameters, including repellency, feeding preference, and survival, were evaluated after exposure to isoplumericin and plumericin, and toxicity was assessed in non-target organisms. Subsequently, a gustatory receptor from *L. longipalpis* was characterized through molecular docking and dynamics to assess ligand interactions and complex stability. These findings clarify molecular and behavioral mechanisms of iridoid interaction with the *L. longipalpis* gustatory system. They provide a foundation for developing sensory-based vector control strategies while ensuring safety for non-target species.

## 2. Results

### 2.1. Preparation of Crude Extract and Isolation of Iridoids

The crude dichloromethane extract was selected for iridoid isolation because of its high content (~90% of the total composition). This was confirmed by GC-MS analysis and supported by literature data [35]. Isoplumericin (29%) and plumericin (60%) were identified as the major constituents, highlighting the potential for large-scale recovery.

**Figure 1:**
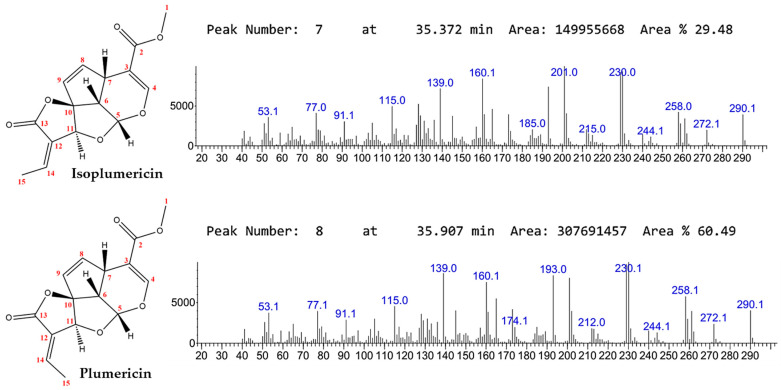
Chemical structures and GC-MS spectrum of isoplumericin and plumericin.

Flash column chromatography under optimized conditions was used to maximize separation. Elution of fractions 3–5 yielded a white crystalline solid with a 68% (*w*/*w*) yield relative to the crude extract. GC-MS analysis (Figure 1) of the pooled fraction revealed a mixture of isoplumericin (41%) and plumericin (59%), confirming the efficiency of the fractionation method. NMR analyses further confirmed the identity of the mixture and were consistent with published data [36,37].

### 2.2. Survival Studies

**Figure 2:**
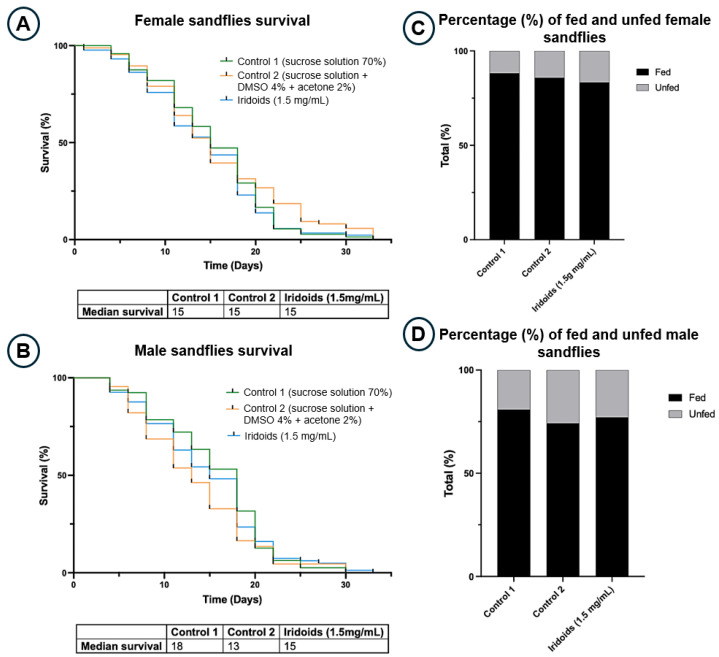
Survival and feeding behavior of adult *L. longipalpis.* (**A**,**B**) Survival curves of males and females exposed to different diets: control 1 (70% sucrose), control 2 (70% sucrose + 4% DMSO + 2% acetone), and iridoid mixture (70% sucrose + solvents + 1.5 mg/mL iridoids). Two biological replicates, *n* = 50/cage. Log-rank test; *p* > 0.05 = no significant difference. (**C**,**D**) Percentage of fed and unfed insects recorded during the survival assay.

The effects of iridoids on the survival of adult *Lutzomyia longipalpis* after pupal emergence were evaluated by incorporating these compounds into sugar-based (sucrose 70%) diets under standardized laboratory conditions. To assess the potential impact of the solvents used for iridoids solubilization, a mixture of 4% dimethyl sulfoxide (DMSO) and 2% acetone (solvents) was used as a diluent control (control 2). The iridoids-containing diet and control 2 were compared with a standard 70% sucrose solution (control 1). The assay was conducted in duplicates, and mean values are presented in Figure 2 (Figure 2A,B).

The male survival graph showed that control group 1 had the highest mean survival (18 individuals), while control group 2 had the lowest (13 individuals). Although this difference was only numerical, the Log-rank (Mantel–Cox) test showed no significant difference between the survival curves (*p* = 0.2098). A similar pattern was observed in females, where mean survival rates were identical across controls 1 and 2, as well as the treated group, with no significant difference (*p* = 0.2721).

During the survival assay, the feeding status of insects was recorded by detecting blue coloration in the intestines, confirming diet ingestion (Figure 2C,D). Analysis of feeding rates indicated that males feed less than females, regardless of the diet. Approximately 77% of males consumed the sugar diet, compared to 85% of females. Feeding across the three diets within the male group showed no statistically significant differences according to the nonparametric Kruskal–Wallis test. Similar results were obtained for females, with no significant differences among the diet groups. Overall, these results indicate that offering a sugar diet containing iridoids did not affect the survival or feeding behavior of sand flies, suggesting that these compounds lack insecticidal or antifeedant effects.

### 2.3. Repellency Studies

**Figure 3:**
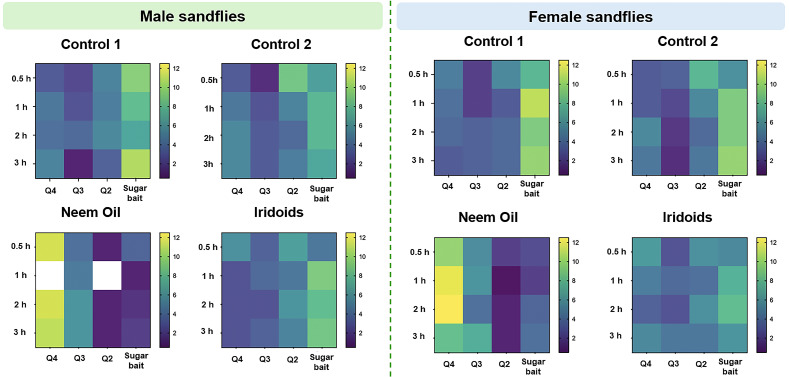
Spatial distribution of L. longipalpis in repellency assays. Treatments tested were control 1 (70% sucrose), control 2 (70% sucrose + 4% DMSO + 2% acetone), iridoid mixture (70% sucrose + solvents + 1.5 mg/mL iridoids), and neem oil (70% sucrose + neem oil). The number of insects was recorded in cage quadrants at 1, 2, and 3 h post-release. Data represent the average of three biological replicates with 20 insects each, with females and males evaluated separately.

Repellency tests were performed in three biological replicates, and results were presented as heat maps illustrating the mean number of insects across replicates (Figure 3). The *x*-axis represents cage quadrants, and the *y*-axis represents observation times. Each intersection corresponds to a quadrant-time combination, color-coded by insects count. Shades closer to yellow indicate higher mean insect counts in the respective quadrant, whereas shades closer to blue indicate lower counts. Blank spaces denote the absence of insects in the corresponding quadrant during the observation period.

### 2.4. Diet Preference Detection

**Figure 4:**
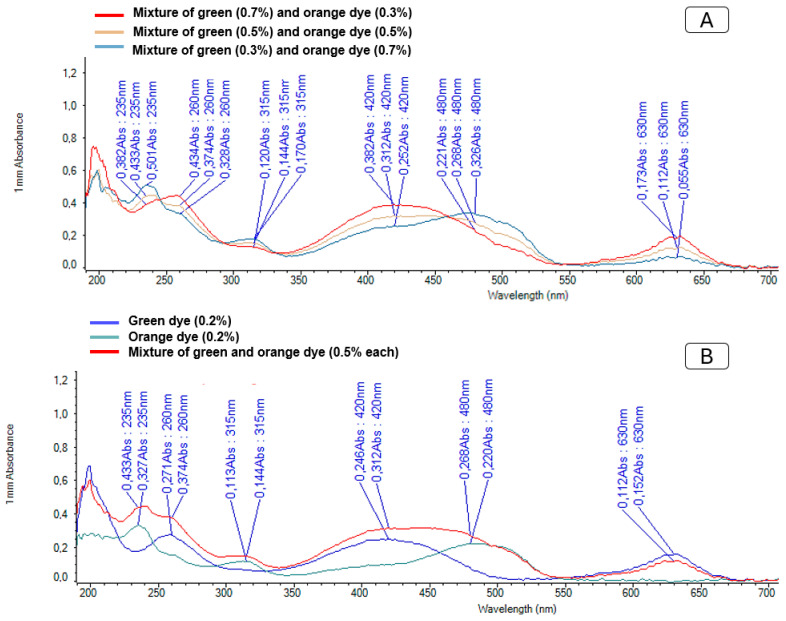
(**A**) Absorption spectra of the green dyes (blue line), orange dyes (green line) and the mixture of both dyes (red line). (**B**) Variations in the absorption curves as a function of the different concentrations of the dyes in the mixtures.

Food dyes were used to distinguish between diets in the preference assays, with orange dye applied to the control diet and green dye to diets containing iridoids. Both diets were offered simultaneously to the sand flies. Food preference was initially assessed by microscopic inspection of intestinal coloration and subsequently confirmed by spectrophotometric analysis using a Nanodrop device (Figure 4). Dyes were selected according to their specific spectral profiles: green dye exhibited absorption peaks at 260, 420, and 630 nm, while orange dye absorbed at 235, 315, and 420 nm.

Spectrophotometric analysis enabled quantification of diet intake even in cases of mixed feeding. Figure 2 shows the spectra obtained for pure dyes and their mixtures at varying concentrations, demonstrating that differences in the spectral curves allow quantitative differentiation of the ingested diets. The high sensitivity confirmed the suitability of this method for accurate detection and quantification of diet preferences.

**Figure 5:**
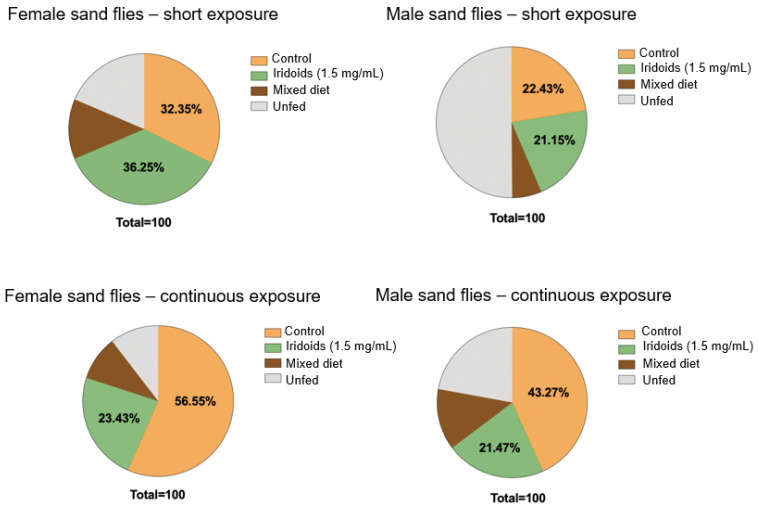
Diet detection based on the absorption of food dyes by females and males of *L. longipalpis* in diet preference experiments conducted under conditions of short and continuous exposure conditions.

The results of the food preference assays are presented in Figure 5. In the short-term exposure experiments, intake was similarly distributed between the control and iridoid-containing diet for both sexes, with no statistically significant differences in consumption proportions. A greater tendency to delay feeding was observed in males, as indicated by a higher proportion of unfed individuals. In contrast, the continuous exposure experiments revealed a distinct pattern: both sexes exhibited a significant preference for the control diet. Females ingested 56.55% of the control diet compared to 23.43% of the iridoid-containing diet, while males consumed 43.27% and 21.47%, respectively. These findings contrasted with the short-term exposure results, indicating that continuous exposure promoted a clear preference for the control diet (orange dye).

### 2.5. Quantification of Sugar Intake

**Figure 6:**
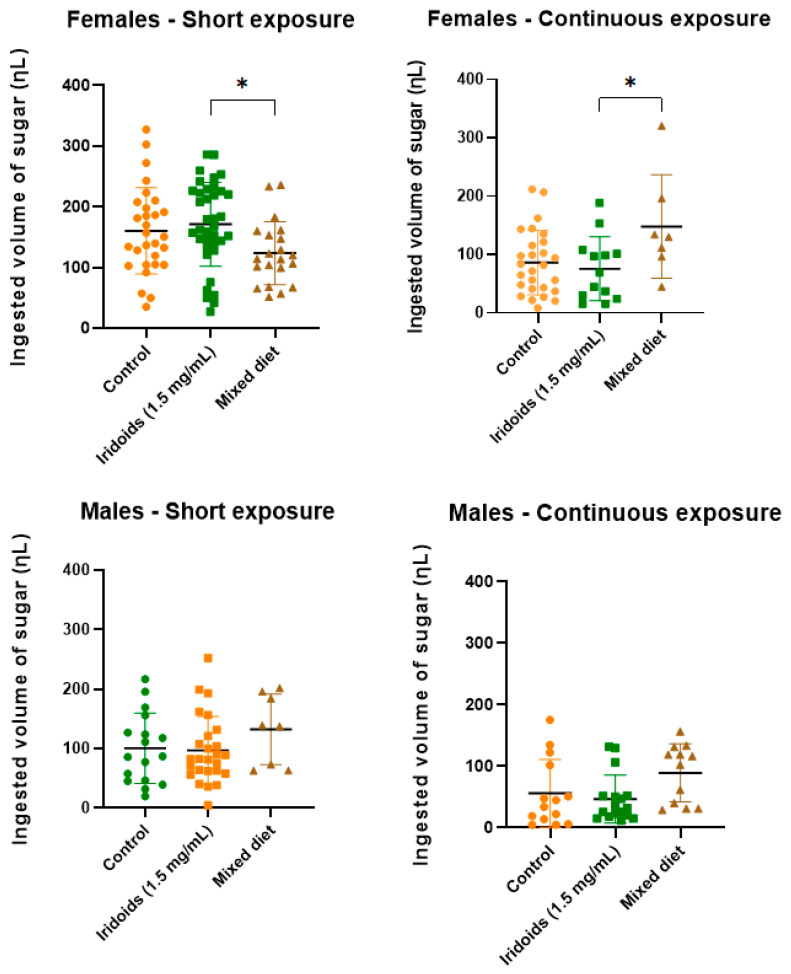
Ingestion (nL) of control (70% sucrose with DMSO 4% + acetone 2%), iridoid mixture (70% sucrose + solvents + 1.5 mg/mL iridoids), and mixed diet (control + iridoids) by adult *L. longipalpis* (males and females). Data are expressed as the mean ± standard error of three biological replicates (30–50 insects per cage per replicate). Statistical significance was assessed by one-way ANOVA followed by Tukey’s multiple comparisons test, with differences considered significant at *p* < 0.05 (*).

Sugar intake was quantified to determine the ingested volume (nL) per insect in each treatment (Figure 6).

In the short exposure experiment, females ingested higher volume from the iridoid-diet, whereas males showed higher intake from the mixed diet. During continuous exposure, the mixed diet was ingested in greater volumes ingested by both sexes. No significant differences in intake were detected between the control and iridoid diets for either sex under both exposure conditions.

In females, significant differences were observed between the iridoid and mixed diets, reflecting higher intake of the iridoid diet during short exposure and higher intake of the mixed diet during continuous exposure. The results also allowed comparison of intake volume between sexes. Overall, intake volumes were in short exposure than in continuous exposure, except for the mixed diet in females, which was higher during continuous exposure. Additionally, significant differences were detected between control and treatment diets across both methodologies, in both sexes.

### 2.6. Acute Toxicity in Non-Target Organism: Drosophila melanogaster

**Figure 7:**
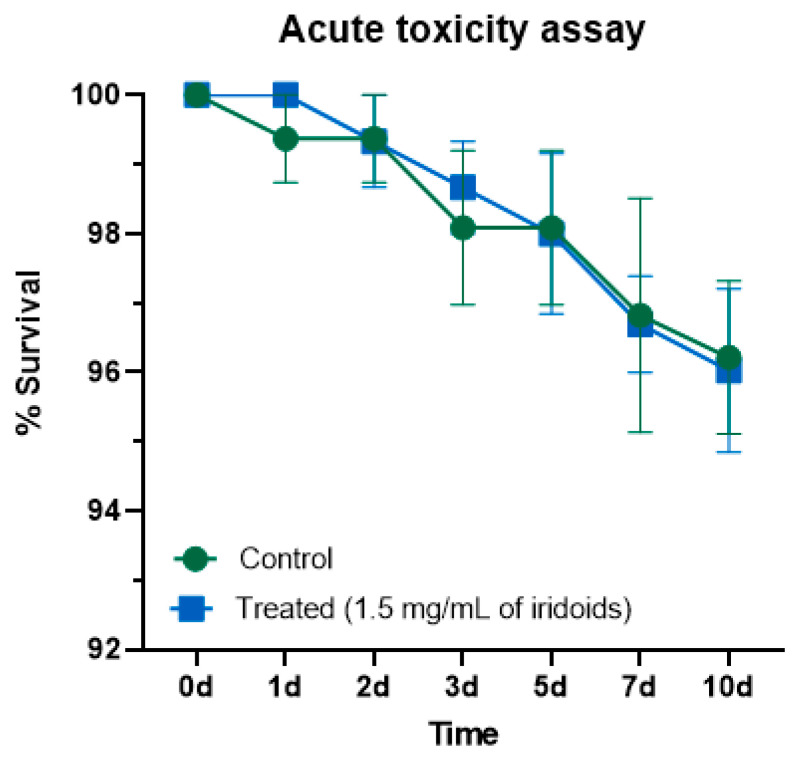
Survival of adult *Drosophila melanogaster* after feeding on an iridoid mixture (1.5 mg/mL) for 10 days to assess acute toxicity. The assay was conducted with four biological replicates. Statistical analysis: Log-rank test (Mantel–Cox); *p* > 0.05 indicates no significant difference.

*Drosophila melanogaster* was employed as a non-target organism to evaluate the acute toxicity of iridoids incorporated into the sugar diet. Adult survival was monitored over a 10-day period (Figure 7). Ingestion of iridoids (1.5 mg/mL) did not result in significant mortality, with both control and treated groups exhibiting approximately 96% survival at the end of the experiment. Statistical analysis indicated no significant difference between the survival curves, confirming the absence of acute toxicity under the tested conditions.

Following the laboratory assays, computational analyses were conducted to investigate the molecular interactions between the A0A1B0CHD5 gustatory receptor from *L. longipalpis* and iridoids.

### 2.7. Homology Modeling, Molecular Docking, and Molecular Dynamics Simulation

#### 2.7.1. Homology Modeling and Quality Assessment

Three distinct computational approaches were employed to generate structural models of the gustatory receptor A0A1B0CHD5. Comparative quality assessment revealed notable differences in model reliability across methodologies (Table 1).

Among the tested platforms, Swiss Model produced the highest-quality structure, with 95.4% of residues in Ramachandran favored regions, surpassing the established >90% criterion for acceptable models according to current validation standards [38,39]. It also achieved a QMEAN6 score of 0.74, compared to 0.73 for Phyre2.2, with both values falling within the high-quality range where larger values indicate better global model quality [40]. However, Swiss Model demonstrated consistent superiority across all evaluated parameters: higher percentage of residues in favored regions (95.4% vs. 94.9%), and more favorable Z-QMEAN (−1.66 vs. −3.00), i.e., closer to zero, indicating better agreement with high-resolution experimental structures. ProSA Z-scores were comparable and within the expected range for proteins of similar size, thus not decisive. In contrast, I-TASSER produced a structurally deficient model, with 80.3% Ramachandran favored residues, below the recommended 90% threshold, and a QMEAN6 score of 0.42, well below the acceptable range for reliable structures. Considering both local stereochemical parameters and global structural quality, the Swiss Model-derived structure was selected as the optimal template for subsequent molecular dynamics simulations and ligand docking studies.

#### 2.7.2. Molecular Docking—Gustatory Receptor A0A1B0CHD5

**Figure 8:**
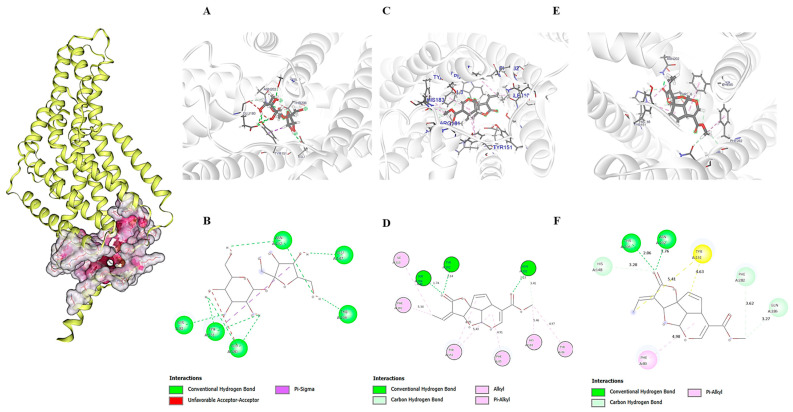
Structural analysis and intermolecular interactions of ligands with GR from *Lutzomyia longipalpis*. Left panel: GR protein structure showing binding cavities (purple/pink regions). (**A**,**B**) Sucrose: (**A**) 3D GR–ligand complex. (**B**) 2D interaction diagram. (**C**,**D**) Isoplumericin: (**C**) 3D binding conformation. (**D**) 2D interaction mapping. (**E**,**F**) Plumericin: (**E**) 3D GR–ligand complex. (**F**) 2D interaction profile.

Molecular docking analysis revealed different binding affinities among the tested compounds (Table 2). Sucrose exhibited the highest ChemLP score of 57.96, interacting with six residues exclusively located in α-helices: Ser82, Glu147, Tyr151, Glu180, Asn202, and Thr206. The interaction profile included conventional hydrogen bonds with Asn202, Glu147, Thr206, and Ser82, π-sigma interactions with Tyr151, and unfavorable acceptor-acceptor contacts with Glu180, indicating an optimized binding site with localized steric constraints (Figure 8). In contrast, isoplumericin showed the most extensive interaction network, involving nine residues, but the lowest binding affinity with a ChemLP score of 47.75. This compound was the only one exhibiting structural diversity in the binding site, interacting with eight residues in α-helices (Tyr302, Asn202, Ser306, Phe282, Tyr151, Phe85, Tyr86, Ile310) and one residue in the loop region (His183). The molecular interactions comprised conventional hydrogen bonds with Tyr302, Asn202, and Ser306, and extensive π-alkyl and alkyl interactions with aromatic and aliphatic residues.

#### 2.7.3. Simulation of the Molecular Dynamics of Ligand–GR Complexes

**Figure 9:**
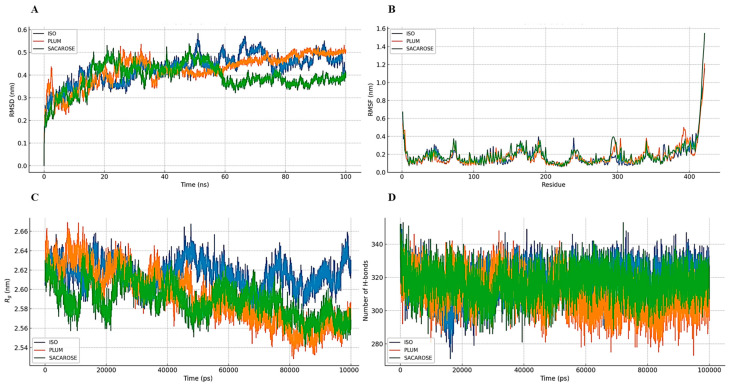
Molecular dynamics analysis of apo-*Lutzomyia longipalpis* gustatory receptor and three ligand–protein complexes (100 ns). (**A**) RMSD of Cα atoms. (**B**) RMSF profile of Cα atoms. (**C**) Radius of gyration (Rg). (**D**) Hydrogen bonds.

Complexes formed between the GR protein and the ligands isoplumericin, plumericin, and sucrose were subjected to 100 ns of molecular dynamics simulations, allowing integrated analysis of structural stability, local flexibility, and conformational properties (Figure 9). Following convergence validation, evaluation of the backbone root mean square deviation (RMSD) confirmed that all complexes maintained stability throughout the 100 ns production runs, with average values calculated using 5 ns block averaging: 0.430 ± 0.064 nm for isoplumericin–GR, 0.426 ± 0.066 nm for plumericin–GR, and 0.391 ± 0.060 nm for sucrose–GR (Figure 9A). These values indicated adequate global conformational stability across the three systems, with sucrose exhibiting smaller fluctuation amplitude, reflecting greater structural rigidity. Residue mean square fluctuation (RMSF) analysis revealed increased mobility in the terminal regions and loop segments, particularly between residues 418–421 (Figure 9B). However, localized flexibility within the binding site was higher in the sucrose–GR complex (local mean of 0.208 nm), followed by plumericin–GR (0.176 nm) and isoplumericin–GR (0.135 nm). This apparent contradiction between the lower global RMSD of sucrose and its greater local flexibility can be explained by the coexistence of global conformational stability with enhanced local plasticity at the active site, a characteristic consistent with adaptive recognition mechanisms.

The distribution of RMSD values supported this interpretation. Isoplumericin showed a unimodal profile centered at 0.35 nm; sucrose exhibited a bimodal distribution with peaks at 0.20 and 0.35 nm; while plumericin displayed a pattern like isoplumericin but with greater dispersion, indicating slightly broader conformational fluctuations. The radius of gyration (Rg) values reinforced this pattern (Figure 9C), showing a slight tendency toward compaction in the iridoid-containing system (2.62 nm for isoplumericin and 2.59 nm for plumericin), compared to sucrose (2.60 nm), demonstrating the subtle influence of ligand topology on protein spatial organization. The number of intramolecular hydrogen bonds (Figure 9D) remained stable across all three systems, fluctuating between 300 and 340 throughout the simulation, supporting maintenance of structural integrity throughout dynamics simulations.

Regarding solvent interactions, the solvent-accessible surface area (SAS) stabilized around 218 nm^2^ for isoplumericin, decreased gradually from 224 to 210 nm^2^ for sucrose, and oscillated subtly between 224.7 and 221.6 nm^2^ for plumericin, with a final average of 218.0 nm^2^. These patterns suggested that iridoids promoted early stabilization of the protein–solvent interface, whereas sucrose induced more pronounced conformational rearrangements over time.

The energy profile of the complexes revealed similar thermodynamic stability values for the iridoids (−7.82 × 10^5^ kJ/mol) that were slightly higher than that of sucrose (−7.80 × 10^5^ kJ/mol), which indicates greater binding affinity for the iridoids.

The overall secondary structure was preserved in all complexes, with an average of 306 structured residues (69.6% of the total 441 residues) in the plumericin–GR system and a comparable distribution in the others, supporting conformational stability. The results confirm that the three ligands formed stable complexes with the GR, each exhibiting a distinct profile of conformational flexibility and molecular recognition.

## 3. Discussion

### 3.1. Characterization and Biological Activity of Isolated Iridoids

The GC-MS chromatogram analysis indicated that isoplumericin had a retention time of 35.3 min, approximately 0.6 min shorter than plumericin’s retention time of 35.9 min, consistent with findings by Ramos et al. [35]. This difference corresponds to the higher relative volatility of isoplumericin among these isomers. Supporting this interpretation, Kupchan et al. [41] reported melting points of 195–198 °C for isoplumericin and 209–212 °C for plumericin. The lower melting point of isoplumericin reflects weaker intermolecular cohesion in the solid state and a higher propensity to transition into the gas phase under identical chromatographic conditions, leading to earlier elution, while plumericin, being less volatile, elutes later.

The selection of dichloromethane extract as the matrix for isolating iridoids was appropriate, as GC-MS analysis indicated that these metabolites account for approximately 90% of the total composition, in agreement with previous reports for the same botanical species [35]. The identification of plumericin (60%) and isoplumericin (29%) in the crude extract highlights the predominance of these two iridoids and indicates strong potential for purification, particularly of plumericin, whose higher abundance favors an increased overall yield. Fractionation by flash column chromatography, performed under optimized conditions, yielded a white crystalline solid from fractions 3 to 5 with a significant recovery of 68% relative to the crude extract.

This observed efficiency demonstrates that the technique is suitable for isolating iridoids with high purity. GC-MS analysis of the combined fraction revealed a binary composition of plumericin (59%) and isoplumericin (41%). Although complete separation of the two isomers, the method effectively concentrated them into a manageable fraction with simplified composition. The outcome is important for subsequent applications, reducing interference from secondary compounds and facilitating additional separation techniques, such as preparative chromatography or fractional crystallization, to obtain individual isomers with higher purity.

Comparative analysis of the proton and carbon NMR spectra of plumericin and isoplumericin (Appendix A) confirmed that both share the same iridoid skeleton, exhibiting very similar chemical shifts and coupling constants for nuclei C-1 to C-9. The most significant differences were concentrated in two structural elements: (i) the acetal center at C-10 and (ii) the α,β-unsaturated portion of the enolactone (C-11 to C-14). At C-10, plumericin showed δ H 5.11 (s) and δ C 80.3, whereas isoplumericin displayed δ H 4.86 (s) and δ C 83.8. The variation between the isomer suggests changes in the electronic and/or stereochemical environment at this center, likely associated with spatial reorientation of vicinal groups, influencing anisotropic effects and the gauche effect on H-10 and C-10 [36].

In the α,β-unsaturated region, plumericin displays a higher chemical shift for H-13 (δ H 7.16) and a lower shift for methyl group, Me-14 (δ H 2.10), while isoplumericin exhibits the reverse pattern (δ H 6.82 and δ H 2.30, respectively). In the proton spectra, C-13 shifts downfield in the isomer, while C-14 shifts upfield. These variations indicate modifications in the geometry of the C-11-C-13 double bond or in the relative positioning of this chain with respect to the lactone ring, influencing the degree of conjugation with the C-12 carbonyl group, which appears slightly more shielded in isoplumericin [36].

Beyond their distinct physicochemical features, both iridoids exhibit remarkable antileishmanial activity reported in the literature. Isoplumericin and plumericin, isolated from *Plumeria bicolor*, showed significant activity with IC_50_ values of 3.17 ± 0.12 μmol and 1.41 ± 0.03 μmol for plumericin, and 7.2 ± 0.08 μmol and 4.1 ± 0.02 μmol for isoplumericin, against promastigote and amastigote forms of *Leishmania donovani*, respectively [34]. Additionally, plumericin obtained from *Allamanda schottii* demonstrated in vitro activity against promastigotes of *L. amazonensis* (IC_50_ = 0.3 μg/mL) and *L. brasiliensis* (IC_50_ = 0.04 μg/mL), representing the most potent antileishmanial compound isolated from this species [33].

### 3.2. Effects on Longevity and Feeding Behavior of L. longipalpis

Longevity studies were conducted to evaluate the toxic effects of the iridoid mixture on *Lutzomyia longipalpis*. Ingestion of the iridoid mixture did not affect survival in sand flies of either sex. In males (Figure 2B), control 1 exhibited the highest mean survival (18 individuals), whereas control 2 showed the lowest (13 individuals). However, Log-rank (Mantel–Cox) statistical analysis indicated no statistically significant differences between survival curves. For females (Figure 2A), mean survival was identical across both control groups and the iridoid-treated group (15 individuals), also without statistically significant differences.

This finding contrasts with the effects reported by Ferreira et al. [42] for mandelonitrile, suggesting that the iridoids tested present a more favorable toxicological profile for the vectors. During the survival assay, diet consumption was also assessed by adding blue dye to the sugar diets, which resulted in visible coloration of the sand flies’ bodies. Quantification of fed individuals (Graphs 1C and 1D) indicated a lower feeding frequency in males (77%) compared to females (85%), irrespective of the diet. Statistical analysis using the nonparametric Kruskal–Wallis test revealed no significant differences in mean food intake among the three diets for either the male or female groups.

The results indicate a natural tendency for females to feed more than males, probably due to higher energy demands for physiological processes such as egg production and reproductive maintenance [43,44]. The absence of significant differences in feeding rates, coupled with the lack of toxicity, confirms that iridoids do not exhibit antifeedant effects and can be safely used.

### 3.3. Evaluation of the Repellent Potential of Iridoids

The results (Figure 3) indicate that the iridoid mixture does not exhibit significant repellent properties under the tested conditions. The behavior response differed markedly from that of the positive control, neem oil (*Azadirachta indica* A. Juss.), confirming the specificity of the repellent effect mediated by compounds active in the sand fly olfactory system [45,46,47]. Neem oil, rich in the limonoids, modulates insect olfactory and gustatory receptors, eliciting repellent and antifeedant effects [48]. In this assay, persistent spatial avoidance was evident, with sand flies predominantly occupying quadrants distant from the treated area throughout the experimental period. The initial concentration of sand flies in the insertion quadrant (Q4) during the iridoid assays corresponded to the exploratory behavior expected after release. The subsequent gradual migration toward the quadrant containing the sugar solution with iridoids (Q1) suggests that the iridoids did not act as negative stimuli nor trigger sensory repulsion through olfactory or gustatory pathways. The heterogeneous distribution observed at the end of the 3 h assay, particularly among females, can be attributed to natural dispersal behavior following initial contact with the food source, a phenomenon documented in sand fly bioecology studies [49].

The absence of repellent behavior in the iridoid-treated groups represents a significant finding for the sensory characterization of these compounds in *Lutzomyia longipalpis*. Some iridoids, such as nepetalactone, nepetalactol, and related compounds found in *Nepeta cataria* and *Actinidia polygama*, are known for their repellent properties towards *Aedes albopictus* [50]. However, the behavioral neutrality observed indicates that the tested iridoids do not induce repellency in *L. longipalpis*, regardless of sex. Even at high concentration (1.5 mg/mL), it remains unclear whether these compounds are neutral to the vector’s sensory receptors or possess low affinity insufficient to elicit adverse behavioral responses. Previous studies on natural compounds demonstrate that both dose and presentation method critically influence the induction of attractive or repellent effects [51]. The present findings therefore suggest sensory neutrality of the tested iridoids at the evaluated concentrations.

Ferreira et al. [52] evaluated synthetic analogs of plant metabolites in sugar solutions and reported that most compounds did not alter sand fly behavior, with only isolated cases showing repellent or attractive effects. Taken together, these observations suggest that behavioral responses are influenced by both the chemical profile of the compounds and the experimental context.

### 3.4. Diet Preference Studies: Temporal Exposure and Choice Behavior

After confirming that the iridoid samples did not exhibit a repellent effect, it became necessary to determine whether the insects preferred a diet containing only 70% sucrose or showed no preference when both diets were offered simultaneously. The identification of the ingested diet in both short and continuous exposure experiments is presented in Figure 5. The food choice assays revealed significant differences between the two experimental models in terms of intake patterns and feeding behavior between sexes. In both experiments, the use of dyes did not influence insect behavior, consistent with previous studies [53,54], which demonstrated that *L. longipalpis* exhibits preferential sensitivity to wavelengths in the ultraviolet range (340 nm) and the green spectrum (520–546 nm).

In the short exposure trials, consumption was similarly distributed between the iridoid diets and the sucrose control, with one-way ANOVA revealing no significant differences between treatments (*p* > 0.05). Within the immediate choice window, no feeding preference between the tested sugars diets was detected. These results indicate the absence of short-term sensory aversion, as the compounds did not trigger immediate taste rejection, thereby preserving palatability. Furthermore, in the same short exposure model, longer feeding latency was recorded in males, consistent with delayed consumption behavior.

In the continuous exposure experiments, an opposite pattern to that observed in short exposure was detected: both females and males exhibited a significant preference for the control sugar diet. Females consumed 56.55% of the control diet compared to 23.43% of the iridoid diet, while males consumed 43.27% versus 21.47%, respectively. The shift in preference indicates that continuous access to food influences decision-making through post-ingestive effects not evident in brief exposure periods. Such effects may involve greater energy yield from sucrose, differences in digestibility and absorption in the midgut, and sensory adaptation integrated with central metabolic regulation. Under these conditions, feeding experiences during the trial appear to shape preference through learning, favoring the diet with the most favorable physiological cost–benefit ratio. The interpretation is consistent with evidence of choice adjustment in insects mediated by internal feedback mechanisms [55].

Therefore, the data align with the literature and indicate that acceptance of iridoids may decrease moderately over time, particularly under conditions that allow continuous gustatory evaluation or metabolic feedback. Such behavior does not preclude the use of iridoids in ingestion-based strategies but highlights the importance of evaluating formulations, concentrations, or natural attractants to ensure adequate acceptance [21,56].

### 3.5. Quantifying Sugar Intake: Dietary Patterns and Physiological Implications

The analysis of sugar intake (Figure 6) demonstrated that exposure time significantly influenced the volume consumed by sand flies in both experimental setups. During short exposure, the highest iridoid intake reached 85% for females and 77% for males. In contrast, during continuous exposure, higher intake of the mixed diet was observed in both sexes. These findings align with expectations, as continuous exposure enables partial digestion of sugars in the intestinal tract of sand flies, resulting in lower quantified volumes. These results indicate that, beyond diet composition, feeding duration significantly influences feeding behavior. Despite within-group variations, comparisons between control and iridoid-treated samples revealed no statistically significant differences for either sex or exposure method, indicating that iridoid addition did not reduce diet palatability. These findings confirm the absence of an antifeedant effect in both in short-term (short exposure) and long-term (continuous exposure) contexts, supporting the potential use of iridoids in strategies that require effective ingestion by vectors.

This profile is desirable for parasite control strategies targeting vectors, as effective ingestion of sugar meals containing iridoids by females is crucial; sugar feeding after a blood meal is essential for the successful colonization and transmission of leishmaniasis parasites [15]. Another important aspect concerns the comparison of ingestion volumes between the two protocols. On average, higher ingestion was recorded in the short exposure trials. In the continuous exposure protocol, progressive digestion and absorption of sugars in the midgut reduce the measurable residual volume at the conclusion of the experiment, which partially explains the lower final quantities. This pattern is consistent with the physiology of feeding and sugar metabolism described for mosquitoes and sand flies [16,57].

### 3.6. Safety Assessment in Non-Target Organisms: Drosophila melanogaster Model

Given the potential environmental exposure to the tested iridoids, evaluation of their possible effects on non-target organisms is required. Traditional in vivo toxicity tests, such as acute assays in mammals, involve high operational costs and raise ethical concerns that often limit their use. As an alternative, simpler experimental models, including roundworms, zebrafish, and fruit flies, are widely employed in biological activity assessments [58]. Among these, *Drosophila melanogaster*, the fruit fly, has become a prominent model for assessing cytotoxicity and toxicity of pesticides [58], nanomaterials [59], and chemical compounds [60]. Its low cost, high reproductive rate, ease of maintenance, and extensive genetic tools have established *Drosophila* as an indispensable organism in basic research [61,62,63].

The evaluation of acute toxicity of iridoids in *Drosophila melanogaster* was conducted to investigate potential effects on non-target organisms, complementing the toxicological characterization of these compounds. Results indicated that continuous ingestion of iridoids at the same concentration used in the sand fly assays (1.5 mg/mL) did not significantly affect adult survival over a 10-day period, with both control and treated groups maintaining approximately 96% survival. The lack of statistically significant differences between survival curves confirmed the absence of acute toxicity of the tested iridoids under the evaluated conditions. These findings contribute to the toxicological profile of isoplumericin and plumericin, indicating low toxicity potential for non-target insects, particularly dipterans phylogenetically related to sand flies. The absence of lethal effects in *D*. *melanogaster* indicated species selectivity, a desirable characteristic for naturally derived compounds [64]. Furthermore, the continuous ingestion conditions of this study further demonstrated that even with continuous exposure, no lethal effects were evident in the test organisms.

### 3.7. Homology Modeling, Molecular Docking, and Molecular Dynamics Simulation

The architecture of insect gustatory receptors has been extensively studied, providing molecular insights into their feeding behavior [65]. Gustatory receptors (GRs), part of a broader family of chemosensory receptors that also include olfactory receptors (ORs) and ionotropic receptors (IRs), have been well characterized in *Drosophila melanogaster*. Among GRs, sugar receptors (SRs), which detect sweet compounds along with other receptors for bitter, salty, and water stimuli, play a central role in feeding decisions, functioning in coordination with proteins and sensory organs [66]. The number of SRs identified in *D. melanogaster* reflects its feeding ecology, which involves the consumption of diverse fruits. Structural studies of fructose receptors have demonstrated highly precise molecular selectivity mechanisms, where the presence or absence of sugar induces specific conformational changes that regulate ion channel opening [65,67].

This study reports the first computational investigation of molecular interactions between the A0A1B0CHD5 gustatory receptor from *L. longipalpis* and iridoids (Figure 8), highlighting its potential role as a molecular sensor capable of discriminating between nutrients and plant secondary metabolites. A high-quality structural model was generated using Swiss-Model, providing a robust computational basis for further analyses. The superior quality of the Swiss-Model prediction corroborates recent findings that demonstrate the effectiveness of homology-based methods when appropriate templates are available [68]. Molecular docking revealed similar ChemPLP affinity scores for sucrose (57.96), plumericin (49.08), and isoplumericin (47.75) (Table 2), suggesting comparable binding capacities of A0A1B0CHD5 for sugars and iridoids. This result agrees with the evidence of integrated processing of taste signals in insects, where gustatory receptor neurons specialized in sugar detection interact with neurons sensitive to bitter compounds to modulate feeding behavior [69].

The 100 ns molecular dynamics simulations, validated through comprehensive convergence analysis, provided valuable insights into the temporal stability of the complexes. The sucrose–GRs complex exhibited a lower mean RMSD (0.391 ± 0.060 nm) compared to the iridoid complexes (isoplumericin: 0.430 ± 0.064 nm; plumericin: 0.426 ± 0.066 nm), suggesting greater conformational stability when bound to the natural sugar. However, RMSD analysis revealed increased local flexibility at the binding site in the sucrose complex relative to the iridoid complexes, indicating that overall stability does not necessarily reflect local rigidity at the binding site (Figure 9).

The correlation between computational data and observed behavioral patterns illustrates the perceptual hierarchy governing feeding decisions in *L. longipalpis*, ranging from initial molecular recognition to integrated final perception. This theoretical framework, proposed by Ruedenauer and coworkers [66], posits that feeding decisions result from multiple processing levels. These include primary sensation (molecular binding to receptors), neurological integration (signal processing from various gustatory receptor neurons), and final perception (incorporation of physiological and metabolic feedback).

During the short exposure assays (3 h), the absence of a clear preference between sucrose and iridoid-containing diets was observed. This result was consistent with the similar affinity scores from docking studies, suggesting limited discrimination at the primary sensation level by initial gustatory receptor recognition. Analyses of RMSD distributions throughout the simulations indicate multiple conformational states of the receptor–ligand complex, possibly corresponding to different stages in the receptor activation cycle. This finding aligns with recent structural insights into the tetrameric architecture of insect gustatory receptors [65,67], where each subunit may adopt distinct conformations during signal transduction. Complex RMSD patterns are recognized as hallmarks of conformational transitions among multiple stable states [70]. The preference observed for the control diet (pure sucrose) during continuous exposure (48 h) reflects the transition from initial sensation to final perception, integrating physiological, neurological, and metabolic factors into a nutritional “percept.” This temporal processing hierarchy implies that essential nutrients are prioritized when sufficient time is available to process post-ingestive feedback from internal receptors and metabolic signals. These factors are not captured by static docking but are fundamental to the nutritional ecology of the species.

The results establish A0A1B0CHD5 as a promising molecular target for the development of rational control strategies for *L. longipalpis* based on modulation of its gustatory system. Structural characterization of this receptor, combined with analyses of conformational dynamics and behavioral assays, indicates that the perception of sucrose versus iridoid compounds involves distinct molecular mechanisms operating on different temporal scales. These findings support the notion that gustatory interferents targeting this receptor could alter feeding preferences, potentially affecting reproductive success and vectorial capacity. Experimental validation through heterologous expression remains necessary to definitively confirm ligand–receptor specificity and computationally predicted electrophysiological responses. Nevertheless, the multidisciplinary approach presented integrating molecular modeling, conformational dynamics, and behavioral ecology provides a robust methodological framework for future investigations. Understanding the molecular mechanisms underlying the perceptual hierarchy in *L. longipalpis* feeding behavior may represent an important step toward the development of eco-rational strategies for controlling this leishmaniasis vector.

## 4. Materials and Methods

### 4.1. Plant Material and Crude Extract Preparation

Roots of *Himatanthus sucuuba* were collected from the Adolpho Ducke Reserve (Amazonas, Brazil), with the specimen deposited under number 5436 in the Herbarium of the Institute of Biological Sciences at the University of Amazonas (Manaus, Amazonas, Brazil). The Brazilian Genetic Patrimony (SISGEN) accession number for this collection is A872C84. The plant material was dried in an air-circulated oven at 35 °C for 24 h and subsequently ground in a knife mill. Extraction was carried out by percolation, first with hexane (Tedia, Fairfield, OH, USA) and subsequently with dichloromethane (Tedia, Fairfield, OH, USA).

### 4.2. Fractionation of the Mixture Isoplumericin and Plumericin from the Dichloromethane Extract

Isolation of iridoids was performed by open column chromatography (400 mm × 30 mm) with 100 g of normal-phase flash silica gel F60 (Silicycle, Quebec City, Quebec, Canada) as the stationary phase. A total of 600 mg of the dichloromethane extract was applied to the column. Elution was initiated with hexane to separate the less polar constituents of the extract, followed by a gradual increase in the polarity of the mobile phase until reaching 100% dichloromethane. This procedure yielded a mixture enriched in the iridoids isoplumericin and plumericin, which was subsequently used in further experiments.

### 4.3. Identification and Characterization of the Iridoids Isoplumericin and Plumericin

#### 4.3.1. Gas Chromatography-Mass Spectrometry (GC-MS)

Isoplumericin and plumericin were identified using Gas Chromatography-Mass Spectrometry (GC-MS). Analyses were performed on Agilent 6890N gas chromatograph (Santa Clara, CA, USA) coupled to an Agilent 5973N quadrupole mass spectrometer (Santa Clara, CA, USA) with electron impact ionization at 70 eV. The system was equipped with a DB-5MS column (30 m × 0.25 mm I.D., 0.25 μm film thickness). Samples (1 μL) were injected in splitless mode, with the injector temperature set to 290 °C and the ion source temperature at 230 °C. The mass spectrometer scanned from 40 to 700 Daltons. The oven temperature was programmed from 70 °C and increased to 350 °C at a rate of 5 °C/min. Helium was used as the carrier gas at a flow rate of 0.5 mL/min. Mass spectra interpretation and compound identification were performed by comparison with the Wiley NBS mass spectral database and with published data [35,37].

#### 4.3.2. Nuclear Magnetic Resonance (NMR)

The iridoids were characterized by Nuclear Magnetic Resonance (NMR) spectroscopy. Experiments were conducted on a Bruker Avance III HD spectrometer (Billerica, MA, USA) operating at 400 MHz for 1H and 100 MHz for 13C, using CDCl_3_ as the solvent and tetramethylsilane (TMS) as the internal standard. Spectra were analyzed, and compounds identification were achieved by comparison with reference data from the literature [36,37].

### 4.4. In Vivo Experiments

#### 4.4.1. Insects

Experiments were conducted with *Lutzomyia longipalpis* sand flies (Jacobina, Bahia, Brazil) maintained under standard laboratory conditions of temperature (26 ± 2 °C) and relative humidity ≥ 70%. Adult sand flies were supplied with a 70% (*w*/*v*) sucrose solution in cotton pads ad libitum, unless otherwise specified in the experimental design.

#### 4.4.2. *Lutzomyia longipalpis* Longevity Curves

Longevity assays were performed following the methodology described by Ferreira et al. [42]. Insects received the iridoids diluted in a solution containing 4% DMSO, 2% acetone, and 70% (*w*/*v*) sucrose solution (Sigma-Aldrich, St. Louis, MO, USA), supplemented with a blue food dye (Arcólor^®^, São Paulo, Brazil). The final concentration of the iridoids was 1.5 mg/mL. Control groups consisted of two treatments: (i) 70% (*w*/*v*) sucrose solution (negative control) and (ii) 70% (*w*/*v*) sucrose solution with 4% DMSO and 2% acetone (vehicle control). All solutions were provided *ad libitum* as drops applied onto Parafilm-lined Petri dishes. Insects were maintained in a BOD incubator under controlled temperature (26 ± 1 °C) and relative humidity (60–70%). Mortality was recorded three times per week, and fresh experimental solutions were supplied every 2–3 days. The assays were performed in two independent biological replicates.

#### 4.4.3. Repellency Tests on Sand Flies

The methodology was adapted from Ferreira et al. [52]. Insects were fasted for 24 h, receiving only distilled water supplied on moistened cotton. Bioassays were conducted in acrylic cages (25.5 × 25.5 × 70.5 cm) divided into four linear quadrants: Q1 (feeding solution end), Q2 and Q3 (intermediate quadrants of 23.5 cm each), and Q4 (insect release end, 25.5 cm). A perforated internal wall separated Q4 from the other quadrants. The feeding solution, either the control (70% sucrose) or the experimental solution containing iridoids (1.5 mg/mL), was placed in Q1. Groups of sand flies (20 males or 20 females) were introduced in quadrant Q4, and their distribution was recorded at 30 min, 1 h, 2 h, and 3 h by counting the number of individuals in each quadrant to assess attraction or repellency.

Each solution was dispensed as 50 drops of 5 μL (total volume 250 μL) in a Petri dish covered with parafilm. Comparative assays included: (i) 70% sucrose (negative control), (ii) sucrose with solvents (vehicle control), and (iii) neem oil in 70% sucrose (positive repellency control). Experiments were conducted under controlled environmental conditions (26 ± 1 °C, 45–60% relative humidity) with three independent biological replicates.

#### 4.4.4. Sugar Intake Experiments

Two experimental methodologies were employed to evaluate feeding preferences of sand flies for the iridoids, differing in the duration of exposure to the experimental diets.

- Short exposure (3 h)

Groups of 30–50 sand flies (males and females evaluated separately) were maintained in cubic cages (13 × 13 × 13 cm) under fasting conditions for 48 h, receiving only distilled water supplied via moistened cotton. On the third day, two diets were offered simultaneously offered for 3 h: (1) control solution containing 70% sucrose, solvents (4% DMSO + 2% acetone), and 5% (*v*/*v*) orange food dye (Arcólor^®^, São Paulo, Brazil); and (2) an experimental solution with the same composition supplemented with the test iridoids (1.5 mg/mL) and 5% (*v*/*v*) green food dye. After exposure, sand flies were dissected, and the contents of the anterior digestive tract (crop) were individually extracted and homogenized in 3 μL of deionized water. Feeding preference was determined by identifying the ingested dye color (green, orange, or a mixed) and expressed as a percentage of the total number of insects analyzed.

- Continuous exposure (48 h)

This methodology was adapted from Ferreira et al. [42]. The experimental protocol was identical to that used for short-term exposure, except that the insects were not subjected to fasting and the diets were provided for 48 h instead of 3 h. Insect maintenance conditions, diet composition, dissections, and crop content analyses followed the procedures previously described.

#### 4.4.5. Detection and Quantification of Sugars from Sugar Intake Experiments

For qualitative detection of the ingested diet, 1 μL of each homogenate obtained from diet preference assays was analyzed using a Nanodrop^®^ 2000C spectrophotometer (Thermo, Waltham, MA, USA). Absorbance was recorded at 260, 420, and 630 nm for the green dye, and 235, 315, and 480 nm for the orange dye.

Quantification of total sugars ingested was performed using a microvolume-adapted phenol-sulfuric acid method based on Dubois et al. [71] and Fox & Robyt [72]. The remaining 2 μL of each homogenate was diluted to 1:25 with 48 μL of deionized water. Aliquots of 0, 5, 10, and 15 μL of the diluted homogenate were transferred to a 96-well microplate, with the final volume adjusted to 25 μL using deionized water. Subsequently, 25 μL of 5% (*v*/*v*) phenol and 125 μL of concentrated sulfuric acid were added to each well. The reaction was incubated at 80 °C for 30 min, and absorbance was measured at 492 nm using a microplate reader.

Sucrose standard curve ranging from 0 to 0.8 mg/mL was used to calculate the sugar concentrations in the samples. Results were expressed as micrograms of sugar per insect. Each sample was analyzed in triplicate.

#### 4.4.6. Non-Target Organism Toxicity

A strain of *Drosophila melanogaster* was obtained from the National Institute of Rural Endemic Diseases (Oswaldo Cruz Foundation, Rio de Janeiro, Brazil). Flies were maintained on a standard feeding diet composed of cornmeal, sucrose, yeast, and agar, with the following formulation: distilled water (500 mL), agar-agar (4 g, Merck, Germany), sucrose (25 g, União, Brazil), cornmeal (30 g, Granfino, Brazil), sodium chloride (0.5 g, Sigma-Aldrich, St. Louis, MO, USA), and dry yeast (10 g, Fleischmann, Brazil). Methylparaben (0.75 g, Proquímicos, Brazil), propionic acid (2 mL, Sigma-Aldrich, St. Louis, MO, USA), and 95% ethanol (3.75 mL, Tedia, Fairfield, OH, USA)) were incorporated as antifungal agents.

To prepare the medium, the dry ingredients (sucrose, agar-agar, cornmeal, sodium chloride, and yeast) were added to boiling water with continuous stirring for approximately 20 min. After cooling the mixture to below 70 °C, methylparaben, propionic acid, and ethanol were incorporated. The medium was then divided into two equal portions: one portion (control) was poured directly into plastic Petri dishes, whereas the second portion was supplemented with the iridoids at a concentration of a 1.5 mg/mL before distribution onto plates. Cultures were maintained under controlled temperature (25 ± 1 °C) and relative humidity (70 ± 10%).

### 4.5. In Silico Analysis

#### 4.5.1. Sequence Retrieval and Homology Modeling

The amino acid sequence of gustatory receptor A0A1B0CHD5 from *Lutzomyia longipalpis* (421 residues) was retrieved from UniProtKB database. Three comparative homology modeling approaches were employed: Swiss Model (https://swissmodel.expasy.org/, accessed on 14 July 2025) [73], I-TASSER (https://zhanggroup.org/I-TASSER/, accessed on 14 July 2025) [74], and Phyre2 (http://www.sbg.bio.ic.ac.uk/phyre2/, accessed on 15 July 2025) [75].

#### 4.5.2. Model Quality Assessment

Structural validation was conducted using multiple tools: Ramachandran plot analysis via PROCHECK (SAVES v6.1; https://saves.mbi.ucla.edu/, accessed on 17 July 2025) [76], global quality assessment using QMEAN6 and Z-scores (Swiss Model Assessment), and independent validation using ProSA-web. The quality thresholds applied were as follows: Ramachandran > 90% favored regions, QMEAN6 > 0.6, and Z-scores > −4.0.

#### 4.5.3. Selection and Construction of 3D Ligand Structures

Isomeric SMILES of the ligands were retrieved from the PubChem database (https://pubchem.ncbi.nlm.nih.gov/, accessed on 11 July 2025). Molecular structures were then generated using ChemDraw Professional version 12.0 (PerkinElmer Informatics, Waltham, MA, USA), and three-dimensional visualization as well as file conversion were performed using the Chem3D extension. Energy minimization was carried out with the MM2 force field until convergence was achieved. The optimized 3D structures were saved in mol2 format for subsequent docking analyses.

#### 4.5.4. Validation of Docking Protocol

Prior to molecular docking simulations, the protocol was validated through redocking experiments with three known ligands: isoplumericin, plumericin, and sucrose. Validation was conducted by docking these compounds against the same gustatory receptor (GR) structure used in the main study. The ability to reproduce reference binding poses was evaluated by calculating the root-mean-square deviation (RMSD) between predicted and reference conformations. RMSD values below 2.0 Å were considered indicated of successful validation in accordance with established docking criteria. The results confirmed the reliability of the docking protocol for subsequent binding pose predictions.

#### 4.5.5. Molecular Docking

Molecular docking simulations were carried out using a two-stage approach combining cavity detection and focused docking. In the first stage, cavity detection guided blind docking was performed with the CB-Dock2 server [77,78] to identify potential binding sites on the protein surface. CB-Dock2 integrates cavity detection algorithms with AutoDock Vina to systematically screen the entire protein surface and rank potential binding sites according to druggability and cavity volume.

Based on the CB-Dock2 results, the coordinates of the highest-ranked binding cavity (X, Y, Z center) were extracted and used to define the search space for focused molecular docking simulations. Subsequent docking calculations were performed using GOLD v2024.3.0 (Cambridge Crystallographic Data Centre) using the ChemPLP scoring function. The binding site was defined as all atoms within a 10.0 Å radius from the coordinates identified by CB-Dock2. Cavity detection was enabled with automatic genetic algorithm parameters, early termination criteria, and hydrogen bonds considered solvent accessible. For each ligand, the top three binding poses were generated with an RMS tolerance of 1.5 Å, and the best poses were selected according to the highest ChemPLP fitness scores.

#### 4.5.6. Analysis of Intermolecular Interactions and Figure Construction

Molecular docking results and receptor–ligand interaction poses were analyzed and visualized using Discovery Studio Visualizer v21.1.0.20298 (BIOVIA, Dassault Systèmes, San Diego, CA, USA; available online: https://www.3ds.com/products-services/biovia/products/molecular-modeling-simulation/biovia-discovery-studio/, accessed on 18 July 2025). The best binding poses, selected based on the highest ChemPLP fitness scores, were evaluated for key intermolecular interactions, including hydrogen bonds, hydrophobic contacts, and electrostatic interactions.

#### 4.5.7. Molecular Dynamic Simulation

To validate the stability of the sucrose–GR, isoplumericin–GR, and plumericin–GR complexes, 100 ns all-atom molecular dynamics simulations were conducted. The computational protocol was executed on the SiBioLead LLP server (https://sibiolead.com/, accessed on 20 July 2025), [79] using the GROMACS package to monitor the temporal evolution of the molecular-dynamic systems. Simulations were performed as a single 100 ns trajectory per complex (no independent replicas).

Each system was solvated in a triclinic box with the Simple Point Charge (SPC) water model, and the OPLS/AA force field was applied to accurately describe biomolecular interactions. Energy minimization consisted of 5000 cycles, followed by ionic neutralization with the addition of 150 mM NaCl (Sigma Aldrich, Brazil) to maintain electroneutrality. Thermodynamic conditions were set at 300 K and 1.0 bar.

System equilibration was assessed through comprehensive monitoring of structural and thermodynamic parameters. Convergence criteria included: (i) backbone RMSD stabilization with plateau formation, (ii) radius of gyration consistency, (iii) maintenance of protein–ligand hydrogen bond occupancy, and (iv) potential energy equilibration. Initial equilibration was performed under NVT/NPT ensembles for 100 ps, followed by extended equilibration until all convergence criteria were satisfied before initiating production runs.

The 100 ns production runs sampled approximately 5000 conformations for statistical analysis. Parameters monitored included: root-mean-square deviation (RMSD) for conformational stability, root-mean-square fluctuation (RMSF) for atomic flexibility, radius of gyration (Rg) for structural compactness, protein–ligand hydrogen bond dynamics, solvent-accessible surface area (SASA), and noncovalent energetics calculated by the MMPBSA methodology. For each trajectory, statistical uncertainties of time-averaged observables were calculated using non-overlapping 5 ns block averaging to account for temporal autocorrelation inherent in molecular dynamics data. This approach treats each block as an independent measurement, providing reliable error estimates for correlated time-series data. Block size was selected to exceed the correlation time of monitored observables, ensuring statistical independence between blocks. Time-averaged values are reported as mean ± standard error (block-averaged) within the defined production window.

### 4.6. Statistical Analysis

Data were analyzed using the software GraphPad Prism version 9.0.2 (https://www.graphpad.com) for Windows. Longevity curves were evaluated through survival analysis test available in the software. Gaussian normality of data distributions was assessed using the D’Agostinho & Pearson test. Samples presenting normal distributions were compared using the parametric unpaired Student’s *t*-test, whereas samples deviating from the normality were analyzed with the nonparametric Mann–Whitney test. For sugar intake quantification, normality of results was assessed by one-way ANOVA followed by Tukey’s multiple comparison test. Results with a *p*-value < 0.05 were considered statistically significant.

## 5. Conclusions

This study provides a multidisciplinary characterization of the iridoids isoplumericin and plumericin in *Lutzomyia longipalpis*, integrating computational and experimental approaches to elucidate their interactions with the vector’s taste system.

Iridoids were isolated with a good yield using a simple and cost-effective methodology. Structural characterization of the gustatory receptor A0A1B0CHD5 through molecular modeling indicated comparable affinities between sucrose and the tested iridoids, consistent with the absence of behavioral discrimination during short-term exposure (3 h). Molecular dynamics simulations revealed distinct conformational mechanisms, with the sucrose–receptor complex displaying greater global stability but increased local flexibility at the binding site.

Behavioral assays demonstrated that the iridoids did not affect *L. longipalpis* survival, showed no repellent properties, and preserved the acceptability of sugar solutions. The preference for the control diet observed only after continuous exposure (48 h) suggests that post-ingestive factors influence taste perception, underscoring the temporal hierarchy of sensory processing in sand flies. Toxicological evaluation in *Drosophila melanogaster* confirmed low toxicity to non-target organisms, reinforcing the safety profile of these compounds.

Overall, the integration of computational, behavioral, and toxicological data provides a solid scientific basis for future investigations into behavioral modulation and taste mechanisms in this leishmaniasis vector, contributing to the advances in the understanding of sensory biology in sand flies.

## Figures and Tables

**Table 1:** Comparative Evaluation of 3D Structural Models Obtained Using Three Different Approaches.

Comparative	% Amino Acids in Ramachandran Plot Regions (PROCHECK)						PROSA
	Favorable	Allowed	Gen. allowed	Nonallowed	Qmean6	Z-QMEAN	*Z*-score
SWISS-MODEL	95.4	4.6	0.0	0.0	0.74	−1.66	−5.8
I-TASSER	80.3	11.6	6.3	1.8	0.42	−12.61	−4.09
Phyre2.2	94.9	4.9	0.3	0.0	0.73	−3.00	−5.91

Model 90. (good)/>95% (excellent); QMEAN6 > 0.6 (acceptable)/>0.7 (good); Z-scores > −4.0 (acceptable range).

**Table 2:** Molecular docking results for ligand–receptor interactions. Binding affinities (ChemLP scores), key interacting residues within the binding site, and redocking root-mean-square deviation (RMSD) values for pose validation are presented for each ligand–receptor complex.

Ligand	ChemLP Score	Key Binding Residues	Redocking RMSD (Å)
Isoplumericin	47.75	Phe85, His183, Tyr86, Tyr151, Ile310, Ser306, Tyr302, Phe282, Asn202	0.132
Plumericin	49.08	His148, Phe85, Thr206, Asn202, Tyr151, Phe282, Gln286	0.051
Sucrose	57.96	Asn202, Glu147, Thr206, Ser82, Tyr151, Glu180	0.627

Plumericin exhibited a ChemLP score of 49.08, interacting with all seven residues exclusively located in α-helices: Phe85, Tyr151, Asn202, Thr206, Phe282, Gln286, and His148. The interaction pattern included conventional hydrogen bonds with Thr206 and Asn202, weak hydrogen bonds with His148, and π-alkyl interactions with the aromatic residues Phe282, Tyr151, and Phe85. Redocking validation confirmed excellent pose reproducibility (RMSD < 0.7 Å) for all analyzed compounds.

## Data Availability

Data is available from the corresponding author upon reasonable request.

## Abbreviations

The following abbreviations are used in this manuscript:

CL	Cutaneous leishmaniasis
MCL	Mucocutaneous leishmaniasis
VL	Visceral leishmaniasis
DMSO	Dimethyl sulfoxide
GC-MS	Gas Chromatograph-Mass Spectrometer
GR	Gustatory Receptor
NMR	Nuclear Magnetic Resonance
RMSD	Root Mean Square Deviation
RMSF	Root Mean Square Fluctuation
SR	Sugar receptor

## Appendix A

The NMR data of ^1^H and ^13^C of plumericin and isoplumericin are disposed below.

*Plumericin*—^1^H-NMR (CDCL_3_, 400 MHz): 5.56 (d, J = 5.59, H-C1), 7.44 (s, H-C3), 4.00 (td, J = 9.6, 2.2, H-C5), 5.64 (dd, J = 3.2, 2.2, H-C6), 6.05 (dd, J = 3.2, 2.2, H-C7), 3.44 (dd, J = 5.9, 3.7, H-C9), 5.11 (s, H-C10), 7.16 (dq, J = 6.5, 1.0, H-C13), 2.1 (dd, J = 6.9, 0.4, H-C14), 3.77 (s, MeOOC). ^13^C-NMR (CDCL_3_, 100 MHz): 102.3 (C1), 166.2 (C2(, 152.7 (C3), 104.6 (C4), 38.4 (C5), 126.4 (C6), 141.1 (C7), 109.4 (C8), 53.7 (C9), 80.3 (C10), 127.4 (C11), 168.2 (C12), 145.3 (C13), 16.1 (C14), 51.7 (C15).

*Isoplumericin*—^1^H-NMR (CDCL_3_, 400 MHz): 5.56 (d, J = 5.59, H-C1), 7.44 (s, H-C3), 3.98 (td, J = 9.6, 2.2, H-C5), 5.63 (dd, J = 3.2, 2.2, H-C6), 6.05 (dd, J = 3.2, 2.2, H-C7), 3.44 (dd, J = 5.9, 3.7, H-C9), 4.86 (s, H-C10), 6.82 (dq, J = 6.5, 1.0, H-C13), 2.30 (dd, J = 7.0, H-C14), 3.76 (s, MeOOC). ^13^C-NMR (CDCL_3_, 100 MHz): 101.7 (C1), 166.2 (C2), 152.7 (C3), 104.1 (C4), 38.2 (C5), 126.1 (C6), 141.2 (C7), 108.9 (C8), 53.7 (C9), 83.8 (C10), 125.8 (C11), 167.4 (C12), 148.2 (C13), 14.8 (C14), 51.6 (C15).
